# Diagnostic and therapeutic yield of a patient-controlled portable EEG device with dry electrodes for home-monitoring neurological outpatients—rationale and protocol of the HOME^ONE^ pilot study

**DOI:** 10.1186/s40814-018-0296-2

**Published:** 2018-05-21

**Authors:** Thomas Neumann, Anne Katrin Baum, Ulrike Baum, Renate Deike, Helmut Feistner, Hermann Hinrichs, Joseph Stokes, Bernt-Peter Robra

**Affiliations:** 1 University Department of Neurology, Otto-von-Guericke-University Magdeburg, Leipziger Str. 44, 39120 Magdeburg, Germany; 2 Leibniz Institute for Neurobiology, Brenneckestraße 6, 39118 Magdeburg, Germany; 3 Chair in Empirical Economics, Otto-von-Guericke-University Magdeburg, Universitätsplatz 2, 39106 Magdeburg, Germany; 4 Institute of Social Medicine and Health Economics, Otto-von-Guericke-University Magdeburg, Leipziger Str. 44, 39120 Magdeburg, Germany; 5 German Center for Neurodegenerative Diseases, Site Magdeburg, Leipziger Str. 44, 39120 Magdeburg, Germany; 6 Forschungscampus STIMULATE, Otto-von-Guericke-University Magdeburg, Sandtorstraße 23, 39106 Magdeburg, Germany

**Keywords:** Portable EEG, Home monitoring, Diagnostic testing, Intra-individual comparison

## Abstract

**Background:**

The HOME^ONE^ study is part of the larger *HOME* project, which aims to provide evidence of diagnostic and therapeutic yield (“change of management”) of a patient-controlled portable EEG device with dry electrodes for the purposes of EEG home-monitoring neurological outpatients.

**Methods:**

The HOME^ONE^ study is the first step in the process of investigating whether outpatient EEG home-monitoring changes the diagnosis and treatment of patients in comparison to conventional EEG (“change of management”). Both EEG devices (conventional and portable) will be systematically compared via a two-phase intra-individual assessment.

In the first phase (pilot study phase), both EEG devices will be used within neurologist practices (all other things being equal). This pilot study (involving 130 patients) will evaluate the technical usability and efficacy of the new portable dry electrode EEG recorder in comparison to conventional EEG devices. Judgements will be based on technical assessments and EEG record examinations of private practitioners and two experienced neurologists (percent of concordant readings and kappa values).

The second phase (feasibility study phase) aims to assess patients’ acceptability and feasibility of the EEG home-monitoring and will provide insights into the extent diagnostic and therapeutic yields can be expected.

For this purpose, a conventional EEG will be recorded in neurologist practices. Thereafter, the practice staff will instruct the patients on how the portable EEG device functions. The patients will subsequently use the devices in their home environment.

The evaluation will compare the before and after documented diagnostic findings and the therapeutic consequences of the private practitioners with those of two experienced neurologists.

**Discussion:**

To the best of our knowledge, this will be the first study of its kind to examine new approaches to diagnosing unclear consciousness disorders or other disorders of the CNS or the cardiovascular system through the use of a patient-controlled portable EEG device with dry electrodes for the purpose of home-monitoring neurological outpatients. If the two phases of the HOME^ONE^ study provide sufficient evidence of diagnostic and therapeutic yields, this would justify (indication-specific) full-scale randomized controlled trials or observational studies.

**Trial registration:**

DRKS DRKS00012685. Registered 9 August 2017, retrospectively registered.

## Background

At present, EEGs can only be recorded with considerable effort over a prolonged period of time under the everyday conditions of the patient. Patients are also unable to place an EEG cap on their own. To address these issues, a portable dry electrode EEG recorder (Fourier One) was developed. This new EEG device, which is integrated into a patient-friendly cap, records brain waves continuously or patient-controlled over a longer period of time. The patient can place the portable EEG device independently without the help of medical staff. With the development of this new EEG device, it is also possible to record brain waves outside medical facilities over a period of time ranging from several hours through to days under everyday conditions in the patient’s home. This results in new possibilities for the differential diagnosis of suspected cases of epilepsy, unclear consciousness disturbances, sleep disturbances, ADHD, or other disorders of the CNS, in contrast to other causes. The use of the new EEG device might also result in changes in the therapeutic management (e.g., changes in medication). These possible changes are summarized under the term “change-of-management” and reflect the therapeutic yield of a patient-controlled EEG home-monitoring. In this regard, the new portable dry electrode EEG recorder is intended to be used for both diagnostic performance and part of the therapeutic management.

Traditional EEG devices are not intended to be used in the home environment of patients, especially due to their immobility. To meet medical and technical requirements and the needs and expectations of patients and professionals, Pinho et al. [1] summarize that EEG systems for outpatient care shall have the following features: “wireless connectivity, dry electrodes, signal resolution, sampling frequency, comfort, portability, signal artefact attenuation, event detection and event prediction” (p. 565). For the purposes of realizing the *HOME* project, we expand the list of necessary features by adding two additional needs: (1) an integrated and structured reporting system and (2) the full coverage of the 10–20 system for electrode placement. There are several EEG devices on the market that meet (at least some of) these demands (e.g., [2–5]); other devices are in the developing process (e.g., [1, 6, 7]). The “Fourier One”^1^ was developed to fulfill all demands and is, to the best of our knowledge, the first that meets all legal requirements (particularly with regard to CE certification) for the use in routine medical care and for home-monitoring.

The Fourier One cap (F1) is a CE-approved^2^ medical device of class IIa. With electrodes located at the EEG typical positions (10–20 system), it has the same technical monitoring and evaluation possibilities as a conventional practice EEG device. Since brain waves can also be recorded under everyday conditions, the cap has a wider range of applications than a conventional practice EEG. The signals are digitized by miniaturized electronics and stored on a chip that is integrated into the cap. The chip is read in the supervising neurological practice. The technical signal quality facilitates the diagnostic assessment of brain waves, which corresponds to the conventional EEGs. A telemedical transmission of the brain waves is generally possible but has yet to be realized.

### Objectives of the *HOME* project and specific objectives of the HOME^ONE^ study

The goal of the *HOME* project is to implement an EEG home-monitoring system with a patient-controlled portable dry electrode EEG recorder for use within the routine care of outpatients (for diagnostic purposes as well as for therapeutic management). The HOME^ONE^ study will be a two-phase (pilot study phase and feasibility study phase) intra-individual comparison that is driven by the following aims:

1. To evaluate the technical usability and efficacy of the new portable dry electrode EEG recorder in comparison to the conventional EEG devices (pilot study phase);

To determine whether the pilot study objectives are met, we will analyze the technical assessments and EEG record examinations of the private practitioners and two experienced neurologists (percent of concordant readings and kappa values). After the technical usability and efficacy are shown, the second HOME^ONE^ study phase will be conducted. This feasibility study will aim to:

(2) Assess patients’ acceptability and the feasibility of the EEG home-monitoring (feasibility study phase);
(3) Provide insights into the extent of expectable diagnostic and therapeutic yields (feasibility study phase).

To evaluate whether this feasibility study was successful, we will analyze the before and after documented diagnostic findings and the therapeutic consequences of the private practitioners and compare them with those of two experienced neurologists. As is the case in the pilot study phase, a technical assessment is also part of the analyses.

If the two phases of the HOME^ONE^ study provide sufficient justification for technical usability, practical feasibility, and diagnostic and therapeutic yields, this will justify (indication-specific) full-scale randomized controlled trials or observational studies.

To realize the goals of the *HOME* project (and the HOME^ONE^ study), which is being performed by the University Department of Neurology Magdeburg, we established a network of practices of neurologists in Saxony-Anhalt, a federal state of Germany.

## Methods/design

### Study phases and settings

The portable EEG monitoring will be performed with patients of neurologist practices. A total of 18 participating specialists from the Saxony-Anhalt region have agreed to the study concept, including an advisory second opinion by the University Department of Neurology Magdeburg (KNEU). The practicing neurologists remain responsible for the medical care of their patients.

The HOME^ONE^ study consists of two phases:

During the *pilot study phase* (first phase), an EEG examination with the portable EEG system (F1) will be carried out with a population of approximately 130 study patients in addition to the conventional EEG approach *in the practices of* private practitioner*s*. As such, two EEG recordings will be performed during one consultation (but in sequence) with all other things being equal. This phase will examine the technical usability and efficacy of the new portable dry electrode EEG recorder and compare it with the different conventional practice EEG devices. It will also draw conclusions about the conformity of the reporting physicians. It is, hence, useful to “calibrate” the physicians, especially those from the KNEU who are responsible for performing the second assessment, before the beginning of the second phase. The inter-rater reliability of EEG evaluations shows kappa values of 0.5 to 0.9 [8–11], depending on the question. As an example, experienced epileptologists feel safe in their findings but have only low inter-rater matches [12]. It cannot be excluded that the additional use of the portable EEG device in neurological practice can already provide a diagnostic, therapeutically useful gain in knowledge.

During the *feasibility study phase* (second phase), the EEG examination will be carried out with approximately 500 study patients. This will commence with a conventional EEG in the doctor’s practice. Subsequently, the neurological practice will introduce the patient to the functioning and handling of the portable EEG device and the necessary accessories. Afterwards, the patient will be able to independently place and remove the portable EEG device and to use it in his or her home environment according to the dispositions of the neurologist.

The doctor in charge will determine the recording time with the cap according to medical criteria. The effective recording time will be included in the empirical evaluation.

The feasibility phase will serve to evaluate patients’ acceptability and feasibility of the EEG home-monitoring process. In addition, this study will aim to generate initial insights into the extent to which EEG home-monitoring achieves the expected diagnostic and therapeutic yield with regard to the use of a patient-controlled, portable EEG device with dry electrodes for home-monitoring of neurological patients.

### Target group, inclusion and exclusion criteria

The study will include patients (male and female of the age of 18 or older) who, according to the judgment of the practicing neurologist, have a suspected or manifest disease giving rise to the medical indication to record an EEG (first or repeated) within the framework of usual statutory care (i.e., billing numbers EBM 16310 or 16311). The private practitioner will be responsible for the selection and the information of the patients.

In both study phases, patients who have to be treated after the first EEG due to hazard prevention will not be included in the study. In addition, patients under the age of 18 will be excluded.

### Patient information and consent

The private practitioners in charge will explain the objectives of the study and the process by which it will be performed to the patients before asking them to sign a consent form in which they agree to the following: portable EEG monitoring, diagnostic second opinion by the University Department of Neurology Magdeburg, data evaluation, and data storage. The signed consent form will remain with the private practitioner. In the event of a possible revocation, the physician shall immediately inform the university department.

### Indication, implementation, and evaluation of EEGs

Before using the conventional EEG, the private practitioner will document the diagnostic question and the therapeutic options. This will serve as the proof for the indication of the conventional EEG. The private practitioner will also document the patient’s medical history.

Both the conventional practice EEG and the portable EEG will be derived in the pilot study phase (first phase) according to uniform standards over 20 min as per the German Society of Clinical Neurophysiology (DGKN) guidelines [13].

In the feasibility study phase (second phase), the EEGs will be derived according to the guidelines of the German Society of Clinical Neurophysiology (DGKN) [13, 14] per the neurologist (possibly also as long-term EEG). The portable EEG will be stored on a memory card that is integrated into the headset. The patients will return with the headset and activity and evaluation sheet, which they will be required to complete.

Subsequently, in both phases of the HOME^ONE^ study, the private practitioner will examine both EEGs using the same standardized assessment sheet.

Both results will be collected by a staff member of KNEU. In the run-up to the second assessment of both EEGs, the Data Trustee will transfer the data from the conventional EEG devices into the standardized format of the portable EEG device and will blind all EEGs. The blinded EEGs will then be examined by two experienced neurologists from KNEU.

### Data analysis and measures

Comparing the two EEG recording methods, the conventional practice EEG cannot be the gold standard to determine the sensitivity, specificity, and predictive values of the portable EEG. The goal that underpins the use of the portable EEG is to gain additional diagnostic insights through extended use. However, in the present state of the method, the portable EEG can remain behind the conventional practice EEG in its diagnostic yield, for example, due to increased artifacts or errors in handling by the patient. As such, it will be necessary to document the technical assessment and the extent to which there is concordance between the two EEGs.

In the pilot study phase, all three assessors will evaluate the technical quality of the EEG records according to the following ratings:

1. The EEG record is unrestrictedly for medical reporting,
2. The use of the EEG record for medical reporting is limited, and
3. The EEG record is not usable for medical reporting.

The evaluation of the technical quality is based on different criteria:

1. The assessor’s overall impression on the EEG record,
2. The frequency of artifacts,
3. Strength of 50 Hz line noise, and
4. Recovery of EEG traces after strong artifacts.

These criteria meet the standards of the International Federation of Clinical Neurophysiology (IFCN) for digital recording of clinical EEG [15].

In addition, all three assessors will document their medical reports for each blinded EEG record. After re-identification of the EEGs and the associated medical reports by the Data Trustee, the concordance (or discordance) will be evaluated. As measurements, we will use concordance rates and kappa values.

In the feasibility study phase, we will use the same approach. However, we will extend it as follows: To share information regarding a possible change of management, the private practitioner will classify the results of the conventional practice EEG according to the possibilities presented in Table 1.

**Table 1:** Action-oriented classification of the findings of the conventional practice EEGs

1A	No action or follow-up appointment only
1B	Targeted referral for further clarification or co-treatment or further treatment
1C	Initiate or change therapy (adjust, switch, settle)

In the same way, they will also classify the results of the portable EEG (see Table 2).

**Table 2:** Action-oriented classification of the results of the portable EEG

2A	No action or follow-up appointment only
2B	Targeted referral for further clarification or co-treatment or further treatment^a^
2C	Initiate or change therapy (adjust, switch, settle)

^a^If clarification or therapeutic clarity can only be established by targeted referral, this clarification is part of the contractual medical care, not the study

In a first simplified evaluation level, the combinations (1A and 2A) as well as ((1B or 1C) will be (2B or 2C)) concordant. This results in Table 3.

**Table 3:** Test assessment of conventional and portable EEGs by the private practitioner

Practice EEG	Portable EEG		Total
	Action (2B, 2C)	No action (2A)	
Action (1B, 1C)	a	b	a + b
No action(1A)	c	d	c + d
Total	a + c	b + d	a + b + c + d

This represents an intra-individual method comparison with a binary result criterion (“change of management”): The case management sequences of the portable EEGs are the same as those of the conventional practice EEGs (a or d), they are beyond (c) or remain behind (b). No decision should be made at this level (c) being “better” than (b).

To develop patients’ acceptability, we will use a questionnaire to measure relevant factors.

In an extended evaluation level, the results of the portable EEG will be more differentiated by means of the possibilities shown in Table 4.

**Table 4:** Differentiation possibilities of the portable EEG results in an extended evaluation level

2A	No action or follow-up appointment only
2B1	Referral to the same specialist as 1B
2B2	Referral to a specialist different than 1B
2C1	Same therapeutic decision as 1C
2C2	Other therapeutic decision than 1C

The combinations of 1A with 2A are concordant with 1B with 2B1 and 1C with 2C1. The other combinations are discordant. Since the expected number of discordances is higher than the previous simplified classification, more cases than stated above will be required.

In the second part of the documentation, the attending physicians will document their gain in case management through the additional portable EEG and determine their disposition for their patient. In doing so, they will decide for themselves which of the possibly discordant EEG findings they consider superior. Since they know both findings and the patient, their two EEG assessments are not independent of each other.

The result of the second opinion in KNEU is subsequently included for the method comparison.

### Sample size calculation

#### Pilot study phase

Because the judgements in this study phase will be mainly based on inter-rater reliability, we will calculate the sample size regarding this issue. According to [16], we will assume a difference between the overall agreement probability and the chance agreement probability of 0.3 and a relative error of 0.3. This will lead to a sample size of 123 subjects. For the pilot study phase, therefore, we expect there to be a duplicate diagnosis for 130 patients.

#### Feasibility study phase

The question of whether one of the two diagnostic procedures indicates for more or fewer patient-targeted referrals or therapies will be tested using a McNemar test.^3^ In a proportion of discordances in the one direction of 0.1 and in the other direction of 0.05 (i.e., 15% of the patients are discordant), and based on the conventional statistical assumptions (error of the first type < 0.05, power > 0.8), a difference between the two procedures can be statistically confirmed with 468 double-diagnosed patients. In the case of 20% discordant patients (with proportions of 0.13 vs. 0.07), 433 patients would be sufficient. For the feasibility study phase, therefore, we expect there to be a duplicate diagnosis for 500 patients.

### Data transfer to the University Department of Neurology (KNEU)

If the procedure described above is followed, the stored EEG data will initially only be available to the initiating physician. According to the protocol, an employee of KNEU will receive the two related EEGs and the assessment form on a data carrier (USB stick) in the practice of the neurologist. In the future, the safe medical network can be employed for this purpose.

### Randomized second assessment by KNEU

The Data Trustee of the KNEU will store the study EEGs and the assessments of the private practitioners separately from the clinic data. KNEU will first evaluate all EEGs according to the technical aspects. Patients with at least one EEG which cannot be assessed technically will not be included in the further method comparison.

Within the framework of the method comparison, KNEU will organize the conciliary second assessment (reference assessment) of the EEGs of each patient by two independent experts. For this purpose, the Data Trustee will pseudonymize the two EEGs of the same patients and randomly divide them before providing both of them to the two specialists who will independently judge them according to the standardized assessment form. After the re-identification of the EEGs by the Data Trustee, any discordance between the outcomes of the two second assessors will be ascertained and jointly (possibly with a mediator) decided whether different dispositions of the further case management result from the comparison of the two related EEGs from the point of view of the KNEU.

**Fig. 1:**
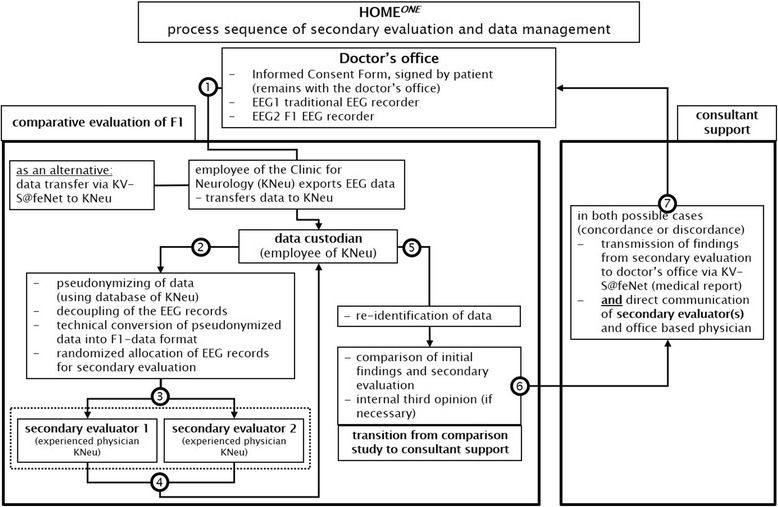
Process sequence of data management, blinded second assessment (KNEU) and conciliary feedback to private practitioner

Subsequently, the findings of the KNEU will be compared with those of the private practitioner. For each patient, the private practitioner will receive conciliary feedback, in urgent cases by telephone (Fig. 1). A final concordance of the three physicians involved will not be required. The professional responsibility for patient management will remain with the practitioner.

In the review of the EEG findings from the practice and the KNEU, there will be two four-fold tables (Table 5).

**Table 5:** EEG assessments by the University Department of Neurology (KNEU) in relation to the previous assessment by the private practitioners

	Practice assessment		
Assessment KNEU	Action (2B, 2C)	No action(2A)	Total
Conventional practice EEG			
Action (1B, 1C)	a	b	a + b
No action (1A)	c	d	c + d
Total	a + c	b + d	a + b + c + d
Portable EEG			
Action (1B, 1C)	a	b	a + b
No action (1A)	c	d	c + d
Total	a + c	b + d	a + b + c + d

The first result criterion will be the proportion and the direction of the cases with discordant disposition of the two EEGs by the private practitioner (Table 3). The second criterion will be the proportion and direction of discordant results in the treatment regimen by specialists of KNEU within the framework of the pseudonymized second assessment.

In this way, it will be possible to determine how the KNEU physicians assess the portable EEG differently from the private practitioners in addition to generating insights into how these two physician groups differ from each other in terms of their assessments of the office-based EEG. Furthermore, it will be possible to observe how the assessments of the consulting physicians from KNEU differ from each other. These assessments will each be quantified as kappa values.

As a third result criterion and final evaluation level of method comparison, the original four-fold table will be prepared again after the consolidation of the first and second assessments (Table 6):

**Table 6:** Consolidated EEG assessments jointly by the University Department of Neurology and by the private practitioners

Practice-EEG^a^	Portable EEG^b^		Total
	Action (2B, 2C)	No action(2A)	
Action (1B, 1C)	a	b	a + b
No action (1A)	c	d	c + d
Total	a + c	b + d	a + b + c + d

^a^ http://powerandsamplesize.com/Calculators/Compare-Paired-Proportions/McNemar-Z-test-2-Sided-Equality

^b^After consolidation of the initial assessment in practice and the second assessment in the University Department of Neurology

### Economics

The physicians involved will care for their patients according to the standard of the statutory health insurance. The study will provide private practitioners with a sufficient number of portable EEG headsets (F1), including the necessary accessories, and a computer suitable for the evaluation, assessment, and documentation of portable EEGs. For the patient-controlled use of the portable EEG devices, the patients will receive, in addition to the EEG headset, a tablet computer that will show the correct function of the device. In addition, the patient will receive a bracelet marker. This will be connected via a radio link to a recording device that is integrated into the headset. This will allow the patient to mark events during the derivation in the recorded EEG curve. The patient will also be provided with clear instructions on how to handle this device by the supervising practice. In this case, the patient will also receive instructions and notes as to which events he can or should mark.

The study-related additional expenses will be funded by two sources. The additional implementation and assessment of the portable EEG will be covered by the participating statutory health insurance funds within the scope of a selective contract.

The participating neurologists will be remunerated for the costs associated with producing the study documentation, which will exceed the standard documentation, from the study fund.

### Privacy and ethics

Participating in the HOME^ONE^ study will be voluntary for physicians and patients. The declaration of consent signed by the patient after the physician has informed him or her of the study details and their possible revocation shall remain with the private practitioner.

The private practices will send the conventional practice EEGs and the additional recorded portable EEGs together (with both assessments) to the University Department of Neurology. The portable EEG may be securely transferred digitally (transfer via a data carrier and later the use of the safe medical network).

Because of the conciliary second opinion, which the patient will have consented to, the University Department of Neurology will represent one of the “co-treating” doctors. It will, therefore, work with patient-identifying data. The second evaluation, which will be performed in the clinic will be performed on pseudonymized data. This will ensure the two related EEGs are assessed independently of each other.

If a patient revokes participation in the study, the private practitioner and the University Department will delete the data collected during the process of the research.

According to professional standards, EEG data are to be safely stored for at least 10 years. The University Department of Neurology can use pseudonymized stored EEGs for the optimization of diagnostic algorithms.

The Ethics Committee of the University Magdeburg has reviewed and approved the project.

## Discussion

The advantage of the described procedure is that it can be immediately transferred and implemented in the outpatient routine care setting. The diagnostic yield will be examined by following a randomized approach. Therapeutic consequences will be legally and practically left to the discretion of the private practitioner. Therefore, no final consensus among the physicians involved in the EEG assessments will be required and, even less, can therapeutic consequences be randomized. To this extent, the medium-term benefits for the patient cannot yet be estimated with the presented design.

The technically extended EEG can lead to an increase in the indication and, thus, to an increase in diagnostic and therapeutic performance. On the other hand, unclear referrals and hospital and sleep-patient stays are likely to reduce. The case documentation will record the planned follow-up dispositions. This will allow a comparative calculation of the statutory health care costs of the two EEG modalities. A medium-term cost-benefit assessment at the level of the health insurance funds or society will not be possible with this design.

It is anticipated that the HOME^ONE^ study can help to increase patients’ acceptability of EEG home-monitoring. We expect the study will shed light on the question of whether such home-monitoring leads to sufficient change of management cases. The feasibility study phase, in particular, will facilitate the identification of particularly relevant medical indications or suspected diagnosis for the use of the EEG home-monitoring. Based on the results, the objective of the *HOME* project is to design and perform indication-specific full-scale randomized controlled trials.

## Abbreviations

ADHD: Attention deficit hyperactivity disorder
CNS: Central nervous system
DGKN: German Society of Clinical Neurophysiology
EBM: Einheitlicher Bewertungsmaßstab (Physicians’ Fee Schedule)
EEG: Electroencephalography
KNEU: University Department of Neurology Magdeburg

## Footnotes

1 Manufacturer: Nielsen Tele Medical GmbH, Magdeburg (http://sites.nielsen.com/telemedical/)

2 According to the CE certificate of the F1 headset, the intended use is specified as follows: The use of the product is indicated for the application in the field of neurology, such as “epilepsy, global brain function diagnosis, and hemispherical asymmetries.”

3
http://powerandsamplesize.com/Calculators/Compare-Paired-Proportions/McNemar-Z-test-2-Sided-Equality
